# Design of the tundra rainfall experiment (TRainEx) to simulate future summer precipitation scenarios

**DOI:** 10.1016/j.mex.2021.101331

**Published:** 2021-04-04

**Authors:** Raleigh Grysko, Elena Plekhanova, Jacqueline Oehri, Sergey V. Karsanaev, Trofim C. Maximov, Gabriela Schaepman-Strub

**Affiliations:** a Department of Evolutionary Biology and Environmental Studies, University of Zurich, Winterthurerstrasse 190, Zurich 8057, Switzerland; b Institute for Biological Problems of the Cryolithozone, Siberian Branch Russian Academy of Sciences, Yakutsk, Russia

**Keywords:** Drought, Precipitation, Automated rainfall manipulation system, Climate extremes, Arctic, Vegetation climate interactions, Harsh environments, Ecosystem functions

## Abstract

The majority of climate models predict severe increases in future temperature and precipitation in the Arctic. Increases in temperature and precipitation can lead to an intensification of the hydrologic cycle that strongly impacts Arctic environmental conditions. In order to investigate effects of future precipitation scenarios on ecosystems, precipitation manipulation experiments are being performed to simulate drought and extreme precipitation conditions. However, most of the existing research so far has been unevenly distributed, primarily focusing on temperate grasslands and woodlands. Despite large changes in the predicted precipitation and potentially high sensitivity of the Arctic tundra ecosystem to these changes, it is among the most understudied ecosystems for precipitation manipulation experiments.

Gherardi and Sala (2013) presented a design for precipitation manipulation experiments that, relative to other methods at the time, was cheap, simplistic, and easily reproducible. In this study, we:

• Present modifications to the original Gherardi and Sala (2013) design that are adapted to cold, harsh conditions, such as those present in the Siberian Arctic tundra.
• Provide a detailed documentation of the improved design.
• Validate our modified experimental design based on the first two years of our experiment.

## Graphical abstract

**Figure:**
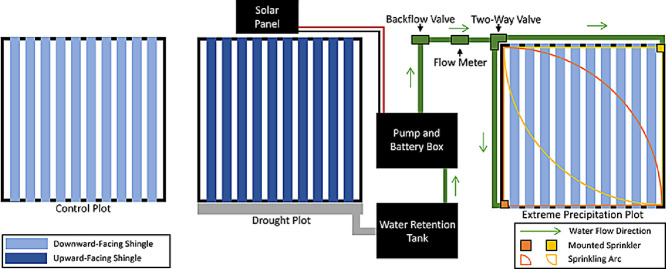

Specifications Table

**Table:** 

Subject Area:	Environmental Science
More specific subject area:	*Rainfall Experiment*
Method name:	*Tundra rainfall experiment (TRainEx)*
Name and reference of original method:	L.A. Gherardi, O.E. Sala, Automated rainfall manipulation system: a reliable and inexpensive tool for ecologists, Ecosphere. 4 (2013) 1–10. 10.1890/ES12-00371.1. A.K. Knapp, M.L. Avolio, C. Beier, C.J.W. Carroll, S.L. Collins, J.S. Dukes, L.H. Fraser, R.J. Griffin-Nolan, D.L. Hoover, A. Jentsch, M.E. Loik, R.P. Phillips, A.K. Post, O.E. Sala, I.J. Slette, L. Yahdjian, M.D. Smith, Pushing precipitation to the extremes in distributed experiments: recommendations for simulating wet and dry years, Glob. Chang. Biol. 23 (2017) 1774–1782. 10.1111/gcb.13504.
Resource availability:	*The data are available in this article.*

## Method details

 

## Introduction

Worldwide changes in temperature and precipitation are among the most consistent predictions made by global climate models [1], [2], [3], [4], [5]. Amplified climatic changes have already been observed in the Arctic, such as an increase in frequency and magnitude of temperature extremes and the subsequent intensification of the global hydrological cycle [6], [7], [8], [9], [10]. Climate change affects energy and water fluxes in the earth system both directly (e.g., via changes in precipitation patterns) and indirectly (e.g., via changes in temperature or moisture demand) [11]. The impact of temperature changes on tundra ecosystems has been widely investigated for over two decades (e.g., Henry and Molau [12]), though relatively little is known about the effects of changes in rainfall, especially in high-latitude Arctic ecosystems. Based on long-term observational data in Greenland, Christensen et al. [13] have recently documented strong effects of extreme weather on ecosystem carbon budgets. Precipitation manipulation experiments are a key tool scientists can use to systematically investigate the impact of future climate extremes on local ecosystems. Simulating expected extreme precipitation and drought on tundra ecosystems in the Arctic is necessary for better understanding climate change effects on biodiversity and ecosystem functions and related feedback between the permafrost and atmosphere. Results can be used to inform vegetation and climate models across the Arctic region.

Precipitation manipulation experiments are typically performed using shelters designed to remove (add) a predetermined amount of precipitation to simulate drought (extreme precipitation) [14,^16^,[31], [32], [33]. Such experiments have been conducted throughout the world [14], though unevenly distributed and with experiments primarily focused around grassland and woodland ecosystems [15,16]. The tundra biome was the third least represented biome, being the study location of only 3.9% of the studies reviewed by Knapp et al. [16], preceded only by the tropical rainforest (3.1%) and dry tropical forest/savanna biomes (2.4%). Precipitation manipulation experiments in ecosystems located in remote regions, such as the Arctic tundra, have been limited by high costs and logistical difficulties related to such studies [14,17]. As a result, most past precipitation manipulation experiments have been limited to five or fewer replicates of experimental treatments [18], [19], [20], [21], [22], [23]. Gherardi and Sala [14] recognized the need for, and subsequently presented, a cheap, simplistic, and easily reproducible precipitation manipulation shelter design. Based on the methods described in Gherardi and Sala [14] and Knapp et al. [16], we present a rainfall manipulation experimental setup adapted and refined to the remote and harsh conditions of Arctic tundra landscapes in this study.

Our automated rainfall experiment established in the Kytalyk National Park (https://www.npkytalyk.ru) in northeast Siberia in 2019 simulates drought and extreme precipitation scenarios by applying the relative rainfall design by Knapp et al. [16] and an adaptation of the rainfall manipulation shelters by Gherardi and Sala [14]. The experiment consists of 30 shelters, with 10 replicates each for drought, extreme precipitation, and control treatments. We first explain our experimental spatial design and rainfall amount calculations (Section 2.), then summarize the Gherardi and Sala [14] setup that we are using as a baseline for our design (Section 3.1.), describe our adapted experimental design and the reasoning behind our choice of precipitation treatment amounts (Section 3.2.), then detail the implementation (including detailed setup and material choices adapted to tundra conditions) and winter proofing of our experiment (Sections 3.2. and 3.3.), explain the automated measurements at our experimental sites (Section 4.), and finally describe how we validated our experiment (Section 5.). The methods presented in this study are applicable to locations of similar geographic, physical, and climatic characteristics.

## Spatial design of experiment and calculation of rainfall amounts for local site conditions

### Experimental study site

**Fig. 1:**
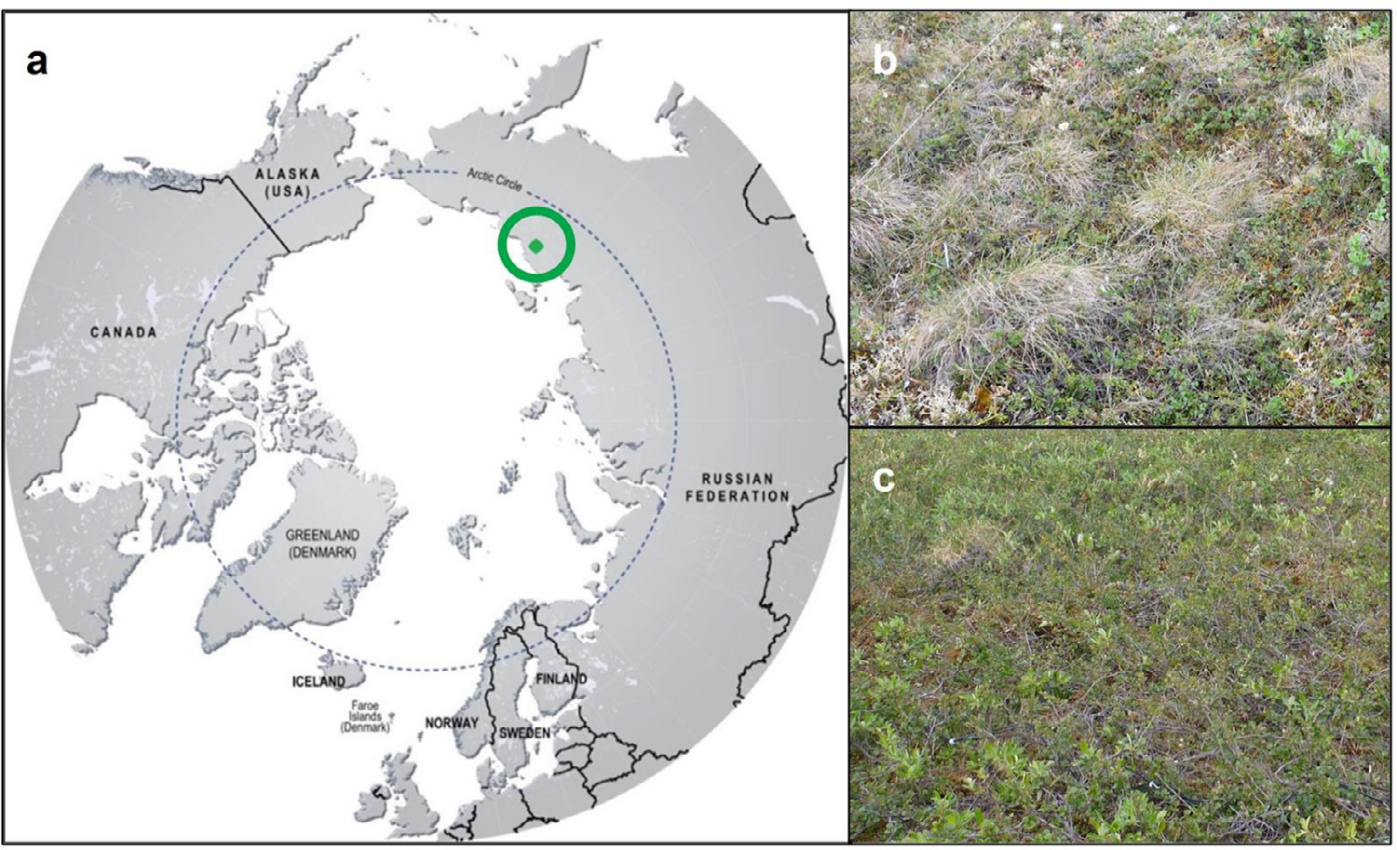
Field site location and vegetation types. (a) Location of Kytalyk National Park, (b) ridge vegetation: tussock-sedge-dominated tundra, (c) drained lakebed vegetation: dwarf-shrub and moss-dominated tundra.

Our field site, the Kytalyk research station, is located in the Kytalyk National Park in the Indigirka lowlands, northeast Siberia (70.83° N, 147.49° E; Fig. 1a). The region belongs to the continuous permafrost zone with an average active layer thickness of 42 cm. The 1981–2010 mean annual temperature of Chokurdakh, a village 28 km southeast of the Kytalyk research station, was -13.4 °C, with a mean July temperature of 10.3 °C. The 1981–2010 Chokurdakh mean annual precipitation was 196 mm, 39% of which fell between June and August [24]. The research area consists of two different morphological units: a drained thaw lake basin surrounded by remnants of Pleistocene yedoma deposits, floodplains, a few thermokarst lakes and drained thermokarst lakes (lakebed); and higher (10–30 m) plateaus with well-drained soils underlain with ice-rich permafrost (ridge). On both locations, the soil consists of silt overlain with 15–30 cm of peat. Morphological features include low and high centered ice wedge polygons and low palsas [25]. Vegetation at the Kytalyk research station primarily consists of dwarf shrub, moss tundra, and tussock sedge (Fig. 1b, c) based on the circumpolar Arctic vegetation map classification [26].

### Spatial design

The vegetation plots for the rainfall treatments were selected across two sites near the Kytalyk research station: one site located on an elevated ridge dominated by tussock-sedge tundra (Fig. 1b) and the second site in a drained thaw lakebed in *Betula nana*-dominated dwarf-shrub tundra (Fig. 1c).

**Fig. 2:**
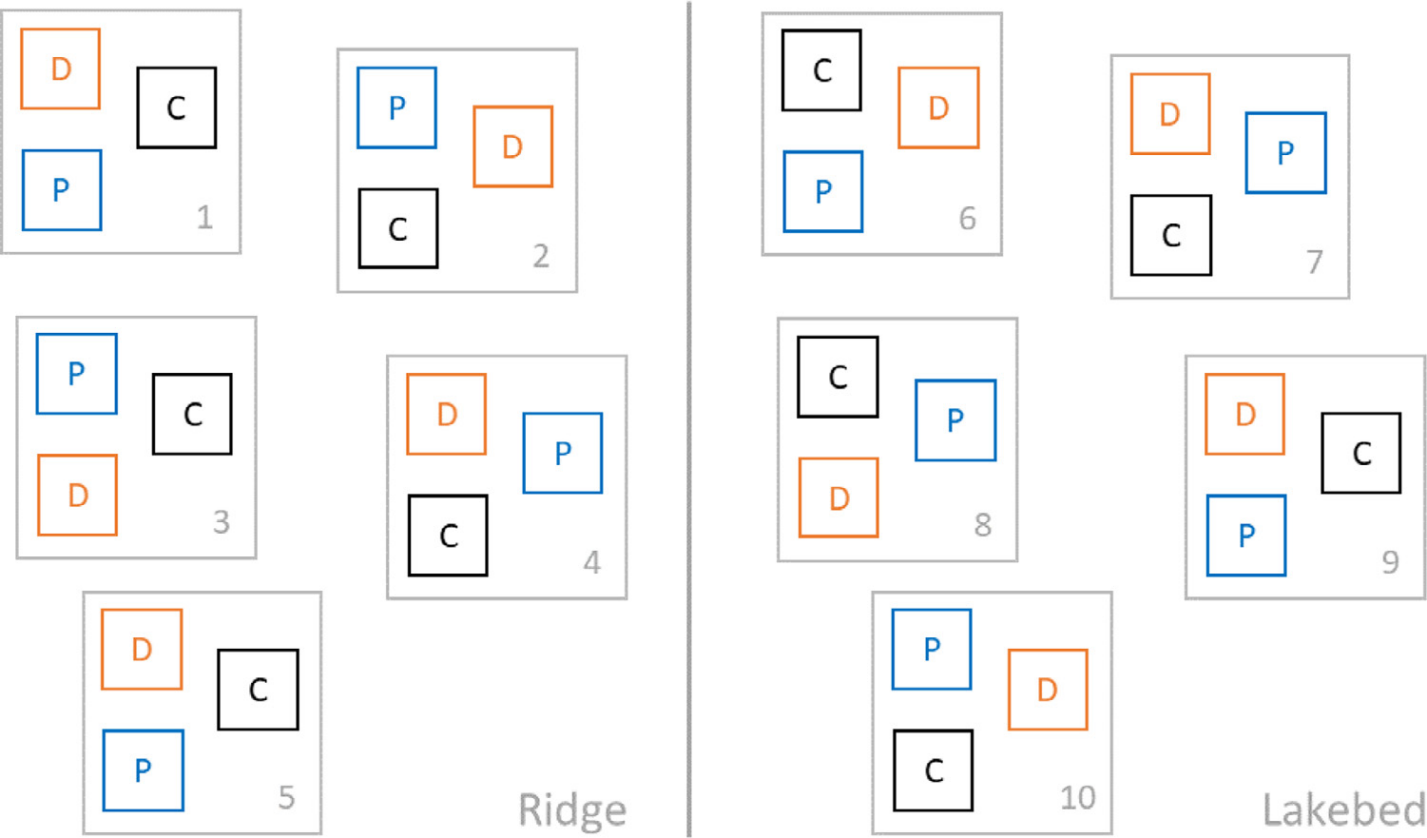
Layout of experimental plots on ridge and drained thaw lakebed sites. The gray squares represent blocks, inner squares represent plots with different treatments: D - drought, P - extreme precipitation, and C - control.

302 x 2 m plots were established in total. Plots were arranged in 10 blocks (5 at each site), with each block consisting of a plot of all three rainfall treatment types: drought, extreme precipitation, and control (Fig. 2). The distance between plots in one block is roughly 2 m.

We chose a 2 x 2 m plot size for our experiment, as indicated by the international protocol for drought treatments for short-statured vegetation < 2 m (https://drought-net.colostate.edu/sites/default/files/the_international_drought_experiment_draft_protocol_v4.pdf). Vegetation in our plots reaches an average height of 12 ± 4 cm.

Blocks and plots were chosen so the vegetation and soil characteristics between plots within blocks are comparable. Specifically, we assessed plant species composition, plant height, specific leaf area, soil pH, soil temperature, and active layer thickness to test pre-treatment conditions. The variance of these characteristics within each block was compared to the variance between each block using ANOVA for normally distributed samples or Kruskal-Wallis test otherwise.

### Calculation of rainfall treatment amounts

Precipitation can be experimentally altered by relative, absolute, or matched methods [16]. Relative amounts for modifying precipitation totals can be defined as adding or removing a predetermined fraction of ambient precipitation (e.g., ± 50% of ambient total). The absolute amount method is defined as removing or adding a specific amount of precipitation, regardless of ambient totals (e.g., ± 50 mm). Matched methods add or remove precipitation relative to ambient amounts to reach a desired precipitation total. Most (roughly 64%) experiments simulating extreme precipitation used the absolute method for determining the magnitude of added precipitation, while roughly 94% of drought simulation experiments determined the magnitude of removed precipitation using a relative approach [16]. Discussions on how to define the precipitation extremes have not subsided [27], [28], [29]. In a recent review, Knapp et al. [16] recommended the relative addition/reduction approach where the treatment levels are matched to historical levels of precipitation variability at a given site because this approach both ensures the comparability between different ecosystems and preserves the frequency and duration of precipitation events observed in naturally-occurring wet and dry years.

**Fig. 3:**
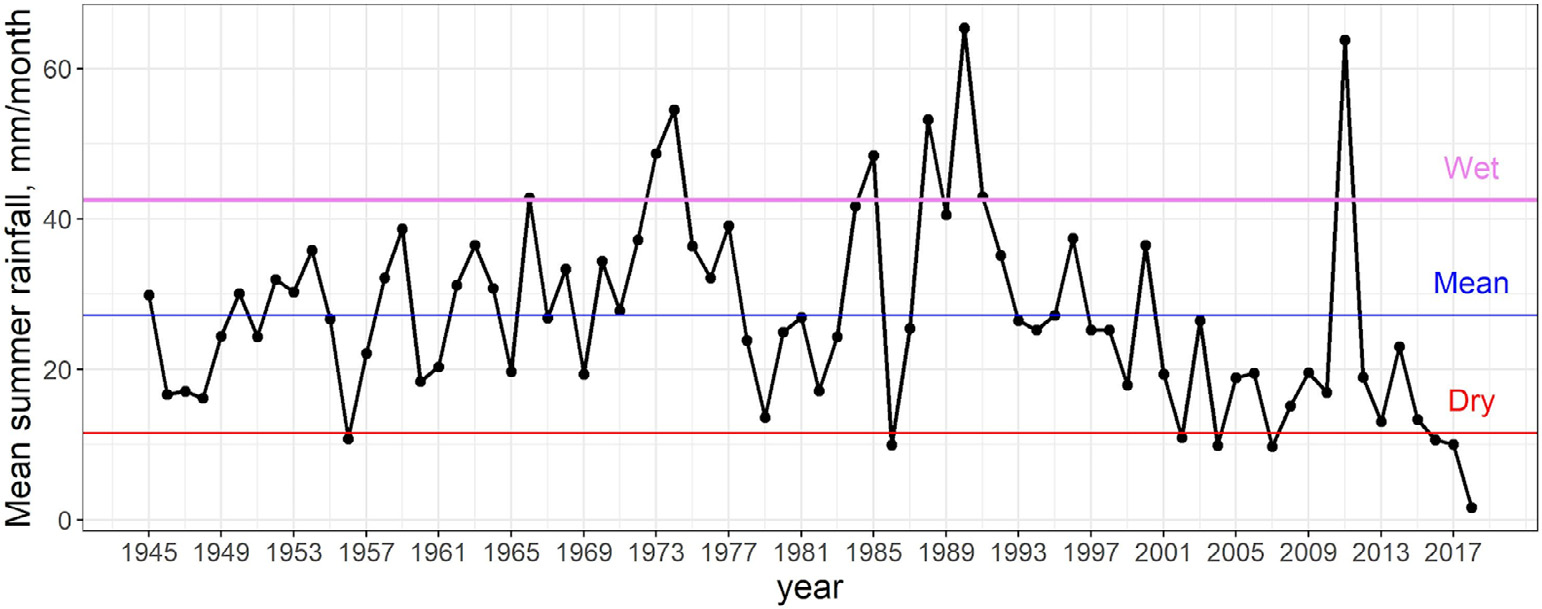
Historical monthly-averaged summer precipitation from Chokurdakh meteorological station. Blue, violet, and red lines correspond to 90th percentile, mean, and 10th percentile, respectively. For interpretation of the color references in this figure legend, the reader is referred to the web version of this article.

Using this recommendation, we chose a relative rainfall treatment based on local rainfall climatology. Extreme precipitation (drought) scenarios were defined as the 90th (10th) percentile of historical precipitation data (Fig. 3). In this study, we used monthly summer (June, July, and August) precipitation data measured from 1944–2018 at the Chokurdakh airport meteorological station, available from the GHCN database, station code: RSM00021946 through NOAA National Climatic Data Center [30]. The 73 year mean summer rainfall was calculated to be 27.2 mm month^−1^, with 10th and 90th percentiles equal to 11.55 mm month^−1^ and 42.48 mm month^−1^, respectively. In other words, a 56% increase in (58% reduction to) average ambient rainfall is required to experimentally simulate extreme precipitation (drought). Rainfall modification percentages were averaged to 57% for ease in rainfall removal and dispersal purposes.

The 42.48 mm month^−1^ rainfall addition also falls into the range of expected precipitation change for year 2100 under the RCP8.5 scenario in the region of the study site: the predicted 40–50% increase of the 1986–2005 average rainfall results to 40.56–43.45 mm month^−1^ [31].

## Automated rainfall manipulation system design and implementation

### Gherardi and Sala [14] system as a baseline

Precipitation manipulation experiments are typically performed using shelters. For example, drought has been simulated using shelters that completely exclude precipitation via permanently closed roofs [[32], [33], [34], [35]], or roofs automatically deployed during precipitation events [36], [37]. However, roofs can also be designed to exclude a certain fraction of precipitation only to simulate specific climate scenarios [38,39]. This approach allows precipitation manipulation to be more realistic relative to complete exclusion shelters. To simulate extreme precipitation, rainfall is added through the use of sprinkler systems, often without any roof shelter.

We base our experimental design on the automated rainfall manipulation system (ARMS) design initially reported in Yahdjian and Sala [39] and further developed by Gherardi and Sala [14]. In their study, Gherardi and Sala [14] describe the implementation and validation of the ARMS, which combines rainout shelters and irrigation shelters into one system. Specifically, the roofs of these shelters capture a relative amount of ambient precipitation that can be adjusted by modifying the roof-shingle density. Captured precipitation is drained off to a water retention tank and then pumped and sprinkled onto the extreme precipitation plot in a quantity equal to the amount intercepted by the paired rainout shelter. A metal support structure was used to support the V-profile acrylic shingles overtop the experimental plot in the ARMS shelters [14]. Their self-made acrylic shingles channeled the intercepted rainfall into a 208 L water retention tank. A float switch installed in the 208 L tank was used to activate the pump after water in the tank reached a predetermined level. The pumped water was distributed over the irrigation plot via two sprinklers installed on opposite corners of the irrigation plot.

Gherardi and Sala [14] confirmed their ARMS design was successful across two study years by validating the experiment using flow meter readings and soil moisture measurements. Flow meter readings were used to create a fitted model of observed precipitation, which was not significantly different when regressed to the expected precipitation and had an intercept not significantly different from zero. As expected, soil moisture was significantly different between treatment types with irrigated (rainout) plots experiencing the highest (lowest) soil moisture values and control plots experiencing intermediate values. Gherardi and Sala [14] were also able to confirm there were no significant differences in photosynthetically active radiation, overall incoming radiation, and temperature between the ARMS and control plots.

### Adapted shelter design for tundra conditions

Contrary to the Gherardi and Sala [14] design, we chose wood as our main construction material of the precipitation shelters due to its low thermal conductivity (particularly important in permafrost regions), being relatively lightweight, availability of material, flexibility during construction (related to uneven ground in tussock tundra), as well as possibility of recycling at the end of the experiment, which is important in remote locations. We used beams with cross-sectional dimensions of 10 x 10 cm for the frame and 5 x 5 cm beams as frame-supporting arms. 5 x 5 cm beams were also used to hold and support the shingles (Supplementary Figs. 1 and 2). Steel L-brackets were used to reinforce the shelter at all wooden connection points. An additional advantage of heavy wooden corner beams was that we could position them on top of the soil and did not need to anchor into the permafrost as the construction was heavy enough to not be disturbed by wind. All shelters were positioned to face towards the on-site prevailing wind direction to further reduce the chance of wind disturbance. Avoiding anchoring the beams also reduced permafrost disturbance and reduced the possibility of structural tilting over time due to potential unequal subsidence from permafrost thaw.

Each shelter was equipped with ten transparent V-profile acrylic glass shingles manufactured by Bröking-Plastex GmbH & Co. KG (model ‘GS 2458’). The acrylic glass used for the shingles is permeable to all photosynthetically active radiation, greater than 90% transparent to wavelengths of 380 to 780 nm, and greater than 80% transparent to the 315 nm UV wavelength [32]. The shingles have a thickness of 3 mm and are 2.5 m in length with flange lengths of 101 mm on either side of the 90° bend in the shingle, meaning each shingle has a hypotenuse length of 142.8 mm. We chose a 2.5 x 2.5 m shelter size to ensure the shelter covers the 2 x 2 m vegetation plots with 25 cm margins where no measurements will be performed due to potential side effects. The total number of shingles necessary to remove the desired 57% of precipitation from the 2.5 x 2.5 m precipitation shelter was calculated using Eq. (1) [32,34].

(1) $$Intercepted\;Precipitation\;\left(\%\right)=\frac{N\times Width\;of\;shingle}{Shelter\;width}\times \;100\%$$

where *N* is the number of shingles.

For the shelter design used in this study, the desired intercepted precipitation is equal to 57%, shingle width equal to 142.8 mm, and shelter width equal to 2500 mm, resulting in a 10 shingle requirement for our selected sheltered area of 2.5 x 2.5 m per treatment plot.

**Fig. 4:**
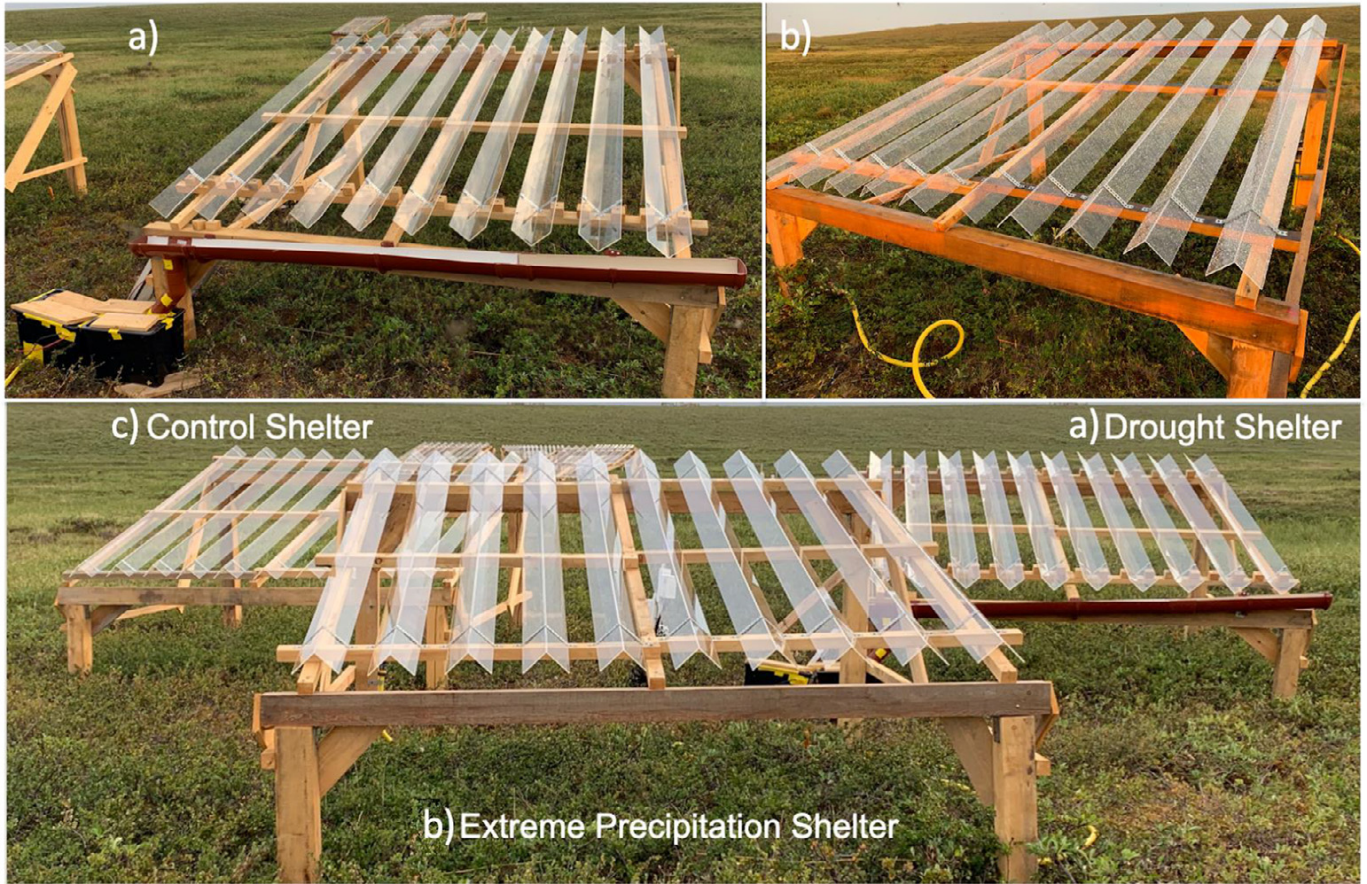
Example of the on-site implementation of the rainfall experiment, highlighting the upward-facing V-profile shingles of the drought shelters (a) and downward-facing V-profile shingles of the extreme precipitation and control treatment shelters (b, c).

**Fig. 5:**
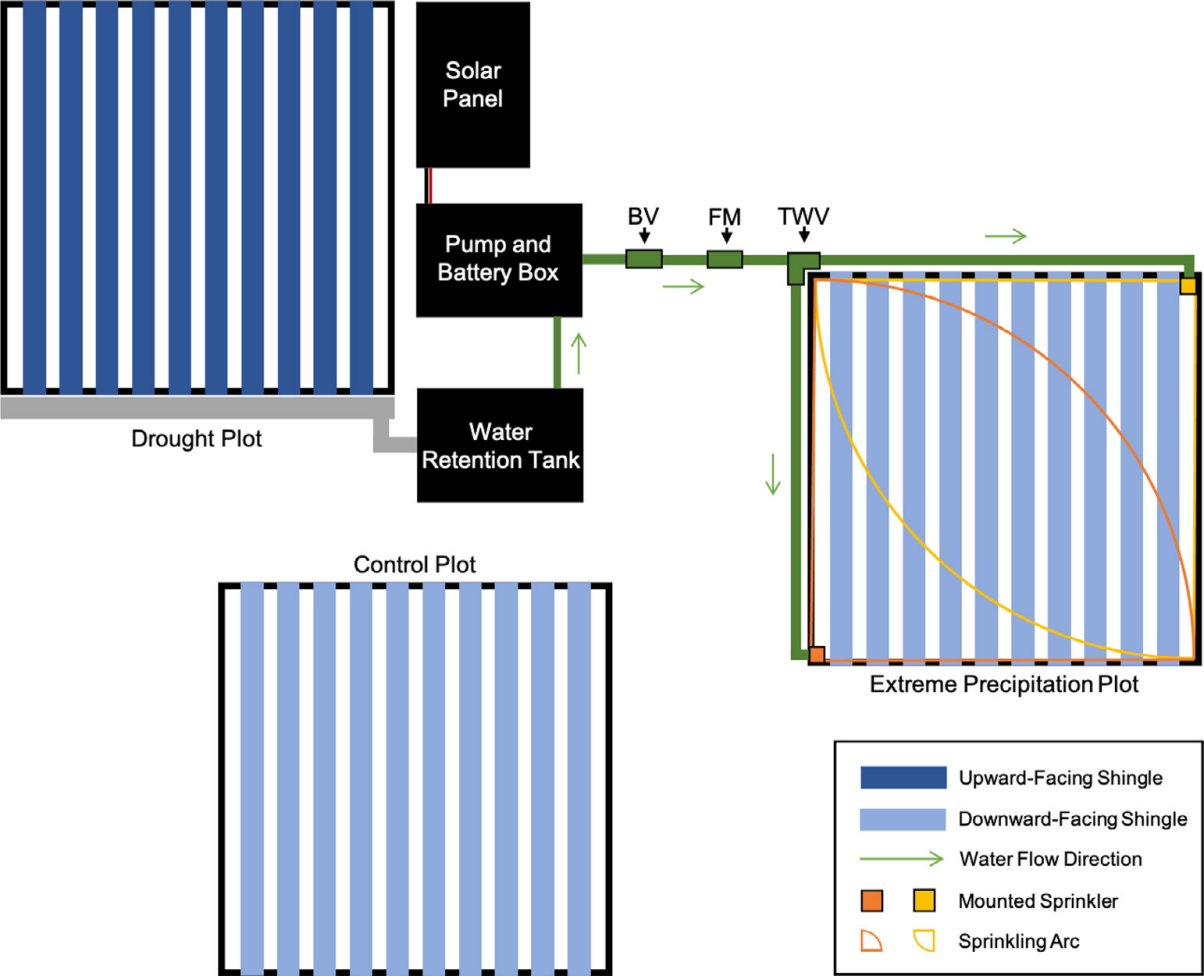
Schematic of our experimental design, including the block layout, electrical components, and water dispersal components. BV, FM, and TWV are defined as Backflow Valve, Flow Meter, and Two-Way Valve, respectively. Experimental design is based on the design originally proposed in Gherardi and Sala [14].

We covered shelters of all treatment types with shingles to ensure comparable wind and light conditions between treatments, which might be influenced by the constructed shelter. This is a modification of the Gherardi and Sala [14] ARMS design, where control and extreme precipitation plots were not covered by shelters. For drought shelters, shingles were oriented with an upward-facing channel created by the 90° bend in the shingle (Fig. 4a). Shingles were oriented downward for the extreme precipitation and control plots to let all incoming rainfall pass through to the plot (Fig. 4b, c). Precipitation intercepted by drought shelter shingles is channeled into a gutter system attached to the drought shelter and then into a water retention tank located at the foot of the shelter (Fig. 4a). An AS88 SPXFlow automatic float switch mounted within the water retention tank is used to activate the 12 V Pacific Hydrostar marine utility pump. Water is pumped from the retention tank to the extreme precipitation plot through a 19 mm diameter, 8.5 m long hose, where it is distributed by two Rain Bird 8-VAN 5 cm sprinklers. To measure the amount of rainfall removed from the drought plots and pumped to the extreme precipitation plots, we installed a flow meter (Gardena Smart flow meter) in the hose between the retention tank and the sprinklers. A backflow valve was installed between the pump and flow meter. The pump is powered by a 12 V 7 Ah battery and charged by a Campbell Scientific SP30 30 W solar panel. The weatherproof ECO10 Phocos Eco Solar Charge Controller is used to regulate the connection between the SP30 solar panel and 12 V 7 Ah battery (Fig. 5).

The pump system is designed to turn on roughly four times per average summer rain day, rain day defined as any day when liquid precipitation occurs, to minimize standing water in the water retention tank between rainfall events. The average rainfall per rain day was calculated using the 73 year historical monthly-averaged summer precipitation data from Chokurdakh meteorological station. Standing water was minimized to avoid algae growth and artificial energy transfer into the soil through the warming of the standing water. See Supplementary Figs. 3 and 4 for additional information about the pump and sprinkler system.

### Winter proofing

**Fig. 6:**
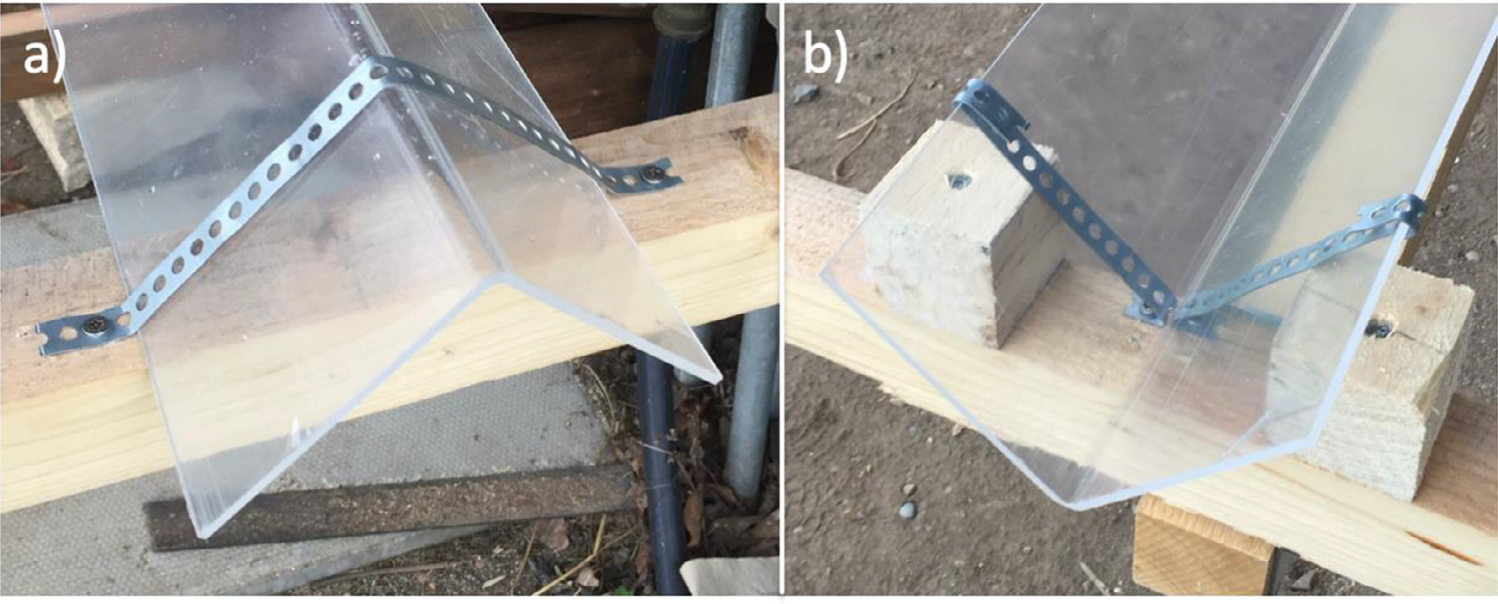
Examples of the flexible metal bands used to secure both the downward-facing shingles (a) and upward-facing shingles (b). Note that small wooden blocks were added for additional support of the upward-facing shingles.

Winter conditions in tundra environments are harsh and the shelter shingles are at risk of being damaged during the winter season due to a combination of low temperatures and snow accumulation on the shingles. As a result, the shingles were designed to be easily attached and removed, which was accomplished using flexible metal bands that secured the shingles to three perpendicular 5 x 5 cm wooden beams (Fig. 6). An alternative and effective method for easily attaching and removing shingles is through the simple use of heavy duty zip ties in place of the flexible metal bands. This, however, is not recommended for long term installation or for implementation in areas with consistently low temperatures.

Once removed, the shingles are stored for the winter in nearby wooden shipping boxes, located far enough away to prevent snow accumulation in the rainfall treatment plots. The remaining shelter structure is left in the field. All other electrical and pump components are also disconnected, removed from the basic shelter structure during the winter season, and stored in an indoor location. It is important to fully disconnect and drain all pump, hose, and sprinkler components to ensure minimal moisture is left in the system during winter storage.

We were able to confirm in late spring 2020 that the above winter proofing measures were effective – the shingles, wooden structures, and all electrical and pump components survived the harsh winter conditions without issue.

## Automated measurements

In order to quantify the precipitation treatment effects, we installed automated sensors for rainfall, air temperature, and soil temperature in our experiment, which is a further expansion to the basic drought experimental design proposed in Gherardi and Sala [14] that only incorporated soil moisture measurements. The data from all sensors are collected using HOBO data loggers (HOBO USB Micro Station Data Logger HS 21; Onset Computer Corporation, Bourne, MA).

### Weather station

We placed a weather station at both the ridge and lakebed sites for our experiment, including a rain gauge and two air temperature and relative humidity sensors at 15 cm and 60 cm above the surface with 10 min measurement frequencies (Table 1). The rain gauge is uninstalled at the end of the growing season, while air temperature and relative humidity measurements are continued during the off season at a lower measurement frequency of 30 min.

**Table 1:** Specifications of installed instruments.

Measurement	Instrument	Unit	Uncertainty
Soil moisture	HOBO S-SMC-M005	m^3^ / m^3^	0.031 m^3^ / m^3^
Soil temperature	HOBO S-TMB-M006	°C	0.2 °C
Air temperature / humidity	HOBO S-THB-M002	°C / %	0.21 °C / 2.5%
Rain gauge	HOBO S-RGF-M002	mm	4% or 0.2 mm

### Soil moisture and temperature sensors

**Fig. 7:**
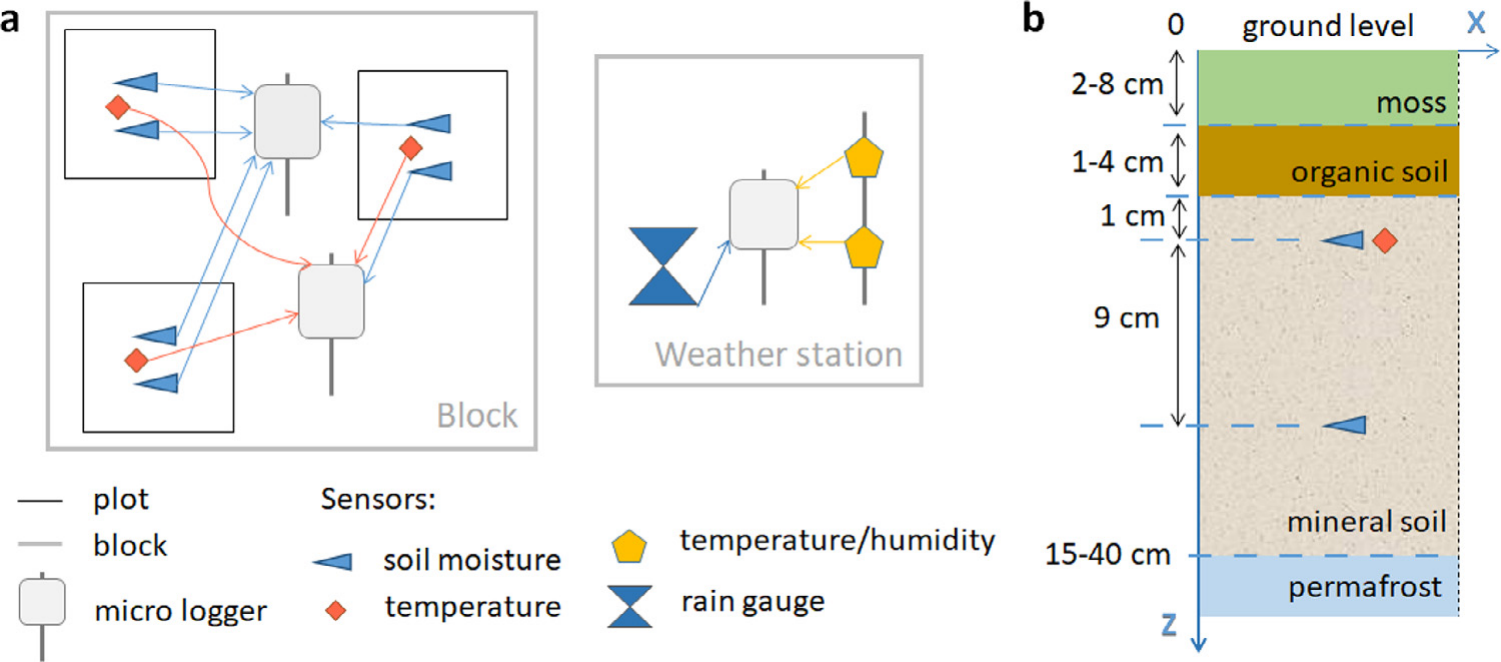
Placement of the soil moisture, soil temperature, air temperature and relative humidity, and rain gauge sensors. (a) connection of sensors to HOBO loggers within a block and a weather station; (b) vertical placement of the soil moisture and temperature sensors in the ground.

In the 0.5 x 0.5 m central area of each of the 30 plots, we placed two soil moisture sensors and one soil temperature sensor (Fig. 7a). We placed soil moisture sensors at 1 and 10 cm depths below the beginning of the mineral soil (Fig. 7b). The temperature sensor was placed near the soil moisture sensor at a 1 cm depth. The frequency of measurements was set to 10 min. All instrument specifications can be found in Table 1.

## Validation of the experimental design

We collected the flow meter data in the summer of 2019 to assess the amount of rainfall received by the extreme precipitation treatment plot, which was then compared with the total amount of precipitation measured by the rain gauges. The average amount of water measured in two months by the 10 flow meters installed near the precipitation plots was 89.4 L with a standard deviation of 6.4 L. This represents the total amount of water added to each of the precipitation plots during this time period. To represent this in mm of added precipitation, we divided this number by the area of the shelter (2.5 x 2.5 m), which resulted in a total of 14.3 ± 1 mm of added rainfall per plot in 2 months. This total was compared to 57% of the total ambient precipitation measured by the rain gauge over the same period (21.6 ± 0.5 mm), which was equal to 12.3 ± 0.3 mm of precipitation.

In 2020, the average amount of water measured in one month was 55.2 L with a standard deviation of 5 L, resulting in 8.8 ± 1 mm of added precipitation. The data from the rain gauge were not available for the same time period, but the weather station in Chokurdakh measured 14.2 mm of precipitation, 57% equal to 8.1 mm, during this period.

We therefore conclude the amounts of precipitation removed from (added to) the plots are very close to the intended drought (extreme precipitation) threshold.

## Direct submission or co-submission

Direct submission.

## Declaration of Competing Interest

The Authors confirm that there are no conflicts of interest.

## Appendix Supplementary materials

Image, application 1
