# Supplementary material for: Design of the tundra rainfall experiment (TRainEx) to simulate future summer precipitation scenarios
Source: MethodsX. 2021 Apr 4;8:101331. doi: 10.1016/j.mex.2021.101331 (PMC8374398; doi:10.1016/j.mex.2021.101331)
Supplement: Supplementary file 1 [file mmc1.docx]

**Supplementary material *and/or* Additional information:**

**Supp. Fig. 1:** Front view of our adaptation of the Gherardi and Sala (2013) automated rainfall manipulation system with wooden piece dimensions. Note the length of the dark purple beams is dependent on the width of the light purple beams.

**Supp. Fig. 2:** Side view of our adaptation of the Gherardi and Sala (2013) automated rainfall manipulation system with wooden piece dimensions.

**Supp. Fig. 3:** Float switch and tubing configuration within the water retention box. The blue arrow indicates the water flow direction

**Supp. Fig. 4:** Wiring configuration within the pump box. Numbering is indicative of connection order – connections 0,a-d do not need to be disconnected between seasonal deployments. Waterproof connections were achieved using 0.5 - 1.5 mm^2^ Scotchloks (manufactured by 3M, part number 314), as indicated by the dark blue cubes. White wires connect to the float switch in the water retention box. Blue arrows on the hose indicate the water flow direction.
